# Predicting disease onset from electronic health records for population health management: a scalable and explainable Deep Learning approach

**DOI:** 10.3389/frai.2023.1287541

**Published:** 2024-01-08

**Authors:** Robert Grout, Rishab Gupta, Ruby Bryant, Mawada A. Elmahgoub, Yijie Li, Khushbakht Irfanullah, Rahul F. Patel, Jake Fawkes, Catherine Inness

**Affiliations:** ^1^Accenture, Leeds, United Kingdom; ^2^Accenture, San Francisco, CA, United States; ^3^Accenture, London, United Kingdom; ^4^Department of Statistics, University of Oxford, Oxford, United Kingdom

**Keywords:** Population Health Management, Electronic Health Records, Deep Learning, chronic disease, Natural Language Processing, disease code embedding

## Abstract

**Introduction:**

The move from a reactive model of care which treats conditions when they arise to a proactive model which intervenes early to prevent adverse healthcare events will benefit from advances in the predictive capabilities of Artificial Intelligence and Machine Learning. This paper investigates the ability of a Deep Learning (DL) approach to predict future disease diagnosis from Electronic Health Records (EHR) for the purposes of Population Health Management.

**Methods:**

In this study, embeddings were created using a Word2Vec algorithm from structured vocabulary commonly used in EHRs e.g., Systematized Nomenclature of Medicine Clinical Terms (SNOMED CT) codes. This study is based on longitudinal medical data from ~50 m patients in the USA. We introduced a novel method of including binned observation values into an embeddings model. We also included novel features associated with wider determinants of health. Patient records comprising these embeddings were then fed to a Bidirectional Gated Recurrent Unit (GRU) model to predict the likelihood of patients developing Type 2 Diabetes Mellitus, Chronic Obstructive Pulmonary Disorder (COPD), Hypertension or experiencing an Acute Myocardial Infarction (MI) in the next 3 years. SHapley Additive exPlanations (SHAP) values were calculated to achieve model explainability.

**Results:**

Increasing the data scope to include binned observations and wider determinants of health was found to improve predictive performance. We achieved an area under the Receiver Operating Characteristic curve value of 0.92 for Diabetes prediction, 0.94 for COPD, 0.92 for Hypertension and 0.94 for MI. The SHAP values showed that the models had learned features known to be associated with these outcomes.

**Discussion:**

The DL approach outlined in this study can identify clinically-relevant features from large-scale EHR data and use these to predict future disease outcomes. This study highlights the promise of DL solutions for identifying patients at future risk of disease and providing clinicians with the means to understand and evaluate the drivers of those predictions.

## 1 Introduction

Health systems across many advanced economies are facing increasing pressure due to a combination of aging populations, increased prevalence of chronic disease, and increasing per capita costs of medical care. In response to these challenges, health policymakers are seeking to move from a reactive model of care which treats illnesses when they arise to a proactive model which intervenes early to prevent adverse healthcare events. The Population Health Management model was developed as an approach to implementing this objective, with the “triple aim” of improving population health, improving the quality of patient experience, and reducing per capita healthcare costs (Berwick et al., 2008). Key to Population Health Management is the use of data; in particular, the ability to identify those patients at risk of future adverse outcomes such as a disease diagnosis (World Health Organization, 2023). Early identification enables health services to support at-risk patients to remain healthier and thus reduce overall consumption of healthcare services (Main et al., 2022). Recent advances in Deep Learning (DL) could be an important contributor to this process, offering the potential to automatically scan large healthcare datasets, detect predictors of morbidity, and model the likely future health outcomes of persons within a population.

An area where DL has the potential to make a dramatic impact is in the management of chronic conditions within populations such as Type 2 Diabetes Mellitus and Chronic Obstructive Pulmonary Disease (COPD). The prevalence of long-term chronic conditions is rising across many industrialized nations, with more than one third of EU citizens reporting living with a chronic condition (Eurostat, 2023). Also rising is the associated cost (Hajat and Stein, 2018; Holman, 2020). In the EU, for example, up to 80% of health care costs are attributable to chronic disease, and in the USA the figure is 86%, with spending predicted to increase further over the coming years (Holman, 2020; Health, 2023). The etiology of these conditions contains modifiable as well as non-modifiable risk factors, so timely forewarning of a likely diagnosis enables persons and healthcare practitioners to take preventative measures that may delay or mitigate onset, improving outcomes and reducing costs.

This paper investigates the ability of a Deep Learning (DL) approach to predict the future diagnosis of long-term chronic conditions (LTCs) and other key adverse health outcomes from Electronic Health Records (EHR). EHR data is increasingly common as frontline digitization efforts have led to increasing adoption of EHR systems (Parasrampuria and Henry, 2019). However, this data presents challenges due to its sparsity and high dimensionality (Zhao et al., 2015). Traditionally, the development of models for disease prediction have placed a heavy reliance on the domain knowledge to define and engineer predictive features from these datasets. This has made predictive disease models expensive and time-consuming to create. It has also meant that clinicians' time has become a rate-limiting factor in the development of new AI solutions, as many countries are experiencing shortages of qualified clinical experts and pressures on their availability is growing. DL has the potential to offer an alternative approach due to its demonstrated ability to learn predictive patterns from data without the need for extensive data preprocessing or feature engineering. Where these DL models were once “black boxes”, advances in algorithms designed to improve their explainability such as SHapley Additive exPlanations (SHAP) offer the promise that the learned features could be analyzed and subjected to clinical validation (Lundberg and Lee, 2017; Markus et al., 2021). This could support the development of clinical trust in AI models and increase adoption (Tonekaboni et al., 2019).

Episodes of care are represented in EHR databases as a series of “concepts”, for example diagnoses, admissions, medications, procedures and observations. These concepts are represented as codes within structured clinical vocabularies which encode medical taxonomies, such as ICD-10, SNOMED, NDC and LOINC. The analogy between these of clinical signifiers and natural language has led researchers to adapt and apply techniques from Natural Language Processing (NLP) to EHR databases, particularly the use of embeddings to represent clinical concepts and the use of Recurrent Neural Networks (RNNs) such as a Gated Recurrent Unit (GRU) or Long Short Term Memory (LSTM) to model the patient history as a sequence of events (Hochreiter and Schmidhuber, 1997; Bahdanau et al., 2014; Pham et al., 2016). Popular methodologies for creating embeddings such as the Word2Vec and Glove have been successfully applied and adapted to this context (Mikolov et al., 2013; Pennington et al., 2014). These methodologies are grounded in the insight from linguistics that the semantic meaning of words may be inferred by the company they keep (Harris, 1954). Thus, the popular Skip-Gram implementation of Word2Vec optimizes vectors for the task of predicting neighboring words given the target word. This has also been demonstrated to be effective in EHR data when “texts” are constructed by placing the codes representing the interactions of individual patients in sequential order. The cosine similarity of medical concept embeddings constructed in this way has been found to cohere with expert opinion in terms of the relatedness of those concepts (Beam et al., 2019).

Multiple methodologies for creating embedding representations of clinical codes have been proposed and tested, and have achieved remarkable success on prediction tasks when compared to baselines. Choi et al. (2016b) built upon the Skip-Gram implementation of word2vec for a clinical context, proposing Med2Vec which they use to learn embedding representations of clinical codes such as diagnoses, medications and procedures (Choi et al., 2016a). They then applied these to predictive modeling tasks, for example the prediction of heart failure, with positive results. Beam et al developed Cui2vec, applying both Word2Vec and Glove to a massive multimodal medical dataset including structured records as well as unstructured notes and journal articles (Beam et al., 2019). Cai et al. (2018) developed a time-aware attention mechanism to augment their Continuous Bag Of Words (CBOW) Word2Vec model in order to capture the differences in the length of time that medical conditions last. Xiang et al. (2019) compared three popular methods of creating embeddings and found Word2Vec to be most effective for predictive modeling tasks.

More recently, following the success of transformer architectures in NLP tasks, researchers have applied these too to the clinical domain. G-BERT combined a medical ontology embedding learned with a graph neural network with a BERT model to predict medical codes in subsequent visits (Shang et al., 2019). Med-BERT also applied the popular BERT architecture which was evaluated on the prediction of diabetic heart failure (Rasmy et al., 2021). BEHRT used an adapted BERT model to create a multilabel classifier able to predict diagnoses in the next 6–12 months from previous diagnosis history (Li et al., 2020). Hi-BEHRT offered an updated version of BEHRT with an improved pre-training strategy capable of modeling longer patient histories, and increased the data scope to include medications, procedures, GP tests, drinking and smoking status as well as binned measurements for BMI and blood pressure (Li et al., 2022). ExBEHRT also extended the feature scope of BEHRT, similarly including observation values for BMI and smoking status as well as procedures, laboratory types, age, race, and gender (Li et al., 2022; Rupp et al., 2023). Wornow et al. (2023) have pointed out however that more needs to be done to prove the practical utility of these foundation models for electronic medical records (FEMRs) to health systems. They emphasize the importance of articulating how such models could fit into clinical workflows, demonstrating their ability to improve predictive performance in contexts where less labeled data is available, and suggesting ways in which they could simplify model deployment.

This paper builds upon this previous research and proposes a simple and scalable methodology by which an FEMR could be implemented for the purposes of Population Health Management. PHM has several features which distinguish it from other areas of clinical practice. Firstly, a key concern of PHM practitioners, in line with the triple aim, is to prevent persons from becoming high users of healthcare services (Stone et al., 2014). This places emphasis on reducing the prevalence of LTCs such as type 2 diabetes, COPD and hypertension as well as acute conditions such as heart attacks that can be mitigated or prevented through lifestyle interventions. Secondly, PHM practitioners are typically engaged with a broader range of determinants of health than other areas of clinical practice; for example, race, gender, economic deprivation, mental health, unmet social care needs and housing status (Buck et al., 2018). These determinants may be captured by many organizations including social and community care providers, local government and third sector organizations, but these may not be standardized in the same way as medical vocabularies and there is likely to be considerable local variation. Developing methodologies that allow models to be developed or easily customized locally within organizations without the financial or human resource to train large domain-general models may therefore be advantageous at least in the short to medium term. Finally, lack of model transparency has been identified as one of the key barriers to the practical implementation of AI solutions for PHM (He et al., 2019). In common with other clinical use cases, PHM requires models to be explainable to clinicians.

The main contributions of this research can be summarized as follows:

1. Developing a simple and effective approach to incorporating an increased data scope, including introducing a novel (to our knowledge) methodology for incorporating complete sets of lab test values into an FEMR and the addition of novel features associated with wider determinants of health.
2. Demonstrating the effectiveness of pre-trained code embeddings to enhance predictive performance for key PHM outcomes where limited data is available, both within and across sub-populations.
3. Investigating whether SHAP could be deployed to improve the explainability of FEMRs to enable clinicians to interrogate the features contributing to the prediction of future outcomes for patients and cohorts.

## 2 Methodology

### 2.1 Data

Retrospective data were obtained from Accenture's AHA platform, which provides structured electronic medical record (EMR) data for ~18% of the US population. This database contains longitudinal medical data from 39 major integrated delivery networks. It represents a broad mix of patients enrolled in privately insured and government-sponsored healthcare programs from a geographically-diverse section of the US population. The database includes information on patient clinical and demographic characteristics, insurance status, healthcare encounters, diagnoses, procedure codes, and associated laboratory values and surgeries, for ~0 million patients. This database has been used as the basis for previous studies, for example (Wertenteil et al., 2019; Sullivan et al., 2021).

### 2.2 Cohort selection

We divided our dataset into an observation window and a prediction window. The observation window was 9 years from 01-01-2010 to 31-12-2018. The prediction window was the final 3 years for which we had data, beginning on 01-01-2018 and ending 31-12-2020. Patients who received a first diagnosis for a given disease after that date were assigned a label of 1; patients who did not were assigned the label 0. Patients who were under the age of 18 at the beginning of the prediction window were excluded from this study, as their risk of the outcomes of interest is extremely low. Patients with very little medical history in the observation window were excluded from the model. This was defined as a record length < 20 total items within the observation window.

On clinical advice, the following additional exclusions were applied on a disease-specific basis:

1. **For diabetes:**
  (a) Patients with a diagnosis for type 1 diabetes
  (b) Patients with a diagnosis for type 2 diabetes prior to 01-01-2018 (pre-existing condition)
  (c) Patients with a diagnosis for Hypoglycemia but no diabetes diagnosis
  (d) Patients with a prescription for anti-diabetic medication or lab values diagnostic of diabetes but no diabetes diagnosis.
2. **For hypertension:**
  (a) Patients with a hypertension diagnosis prior to 01-01-2018 (pre-existing condition)
  (b) Patients with a prescription for anti-hypertensive medications but no hypertension diagnosis.
3. **For COPD:**
  (a) Patients with a COPD diagnosis prior to 01-01-2018 (pre-existing condition)
  (b) Patients with a prescription for medications commonly used to treat COPD but no COPD diagnosis.

In assessing the risk of Myocardial Infarction (MI), individuals with prior diagnoses or on heart disease medications were retained in the dataset. This is because MI is an acute condition and repeated heart attacks are possible. The disease and medication codes used for cohort definition are provided in Table 1. The definitions included all descendants of these codes.

**Table 1:** Codes used for defining disease cohorts.

**Disease**	**Cohort definition (SNOMED-CT)**	**Medications (ATC)**
Type 2 diabetes mellitus	44054006	A10 (drugs used in diabetes)
Essential hypertension	59621000	C02 (antihypertensives)
		C03 (diuretics)
		C07 (beta blocking agents)
		C08 (calcium channel blockers)
		C09 (agents acting on the renin-angiotensin system)
Chronic obstructive pulmonary disease (COPD)	13645005	R03 (drugs for obstructive airway diseases)
Acute myocardial infarction (MI)	57054005	N/A—we predicted any Acute MI rather than a first Acute MI. Patients with previous heart attacks or prescriptions for heart disease medications were not excluded

Our dataset undoubtedly contained patients whose medical history was truncated due to having dropped out of the dataset, either by switching insurer or by leaving the area. This could be expected to impact the performance of our models, as there would be some patients who did experience the adverse outcomes of interest who were labeled as class 0 instead of class 1 because, for example, they moved away and received their diagnosis in another area. However, in contrast to other studies (Li et al., 2020), the decision was made not to attempt to remove these patients from our dataset for several reasons. Firstly, there was no reliable way to distinguish patients who had no medical history because they had dropped out of our dataset from patients who had no medical history simply because they had not visited a doctor during the prediction window. This is due to the nature of medical records—they are only created when patients interact with clinicians, and this is often prompted by illness. Gaps in a patient's medical history could indicate missing data, lack of insurance, or simply good health. Secondly, including only patients who had complete records spanning multiple years would bias the dataset in favor of those more likely to interact regularly with clinicians, who would tend to be wealthier and privately insured. This would have the effect of reducing the number of samples in the dataset from other demographics and therefore potentially reducing the fairness of the model. Thirdly, even a few items of medical history could be enough to indicate a relatively increased risk of disease. For example, a patient with limited items in their medical history that are strongly associated with a predicted disease outcome would be expected to be given an elevated risk by the model, even if it would be difficult to correctly classify them. For our PHM use case, we considered the ability of a model to stratify the population according to relative risk to be more important than correct prediction of class labels at a given threshold.

### 2.3 Preprocessing records

The clinical concepts included in our model were diagnoses, procedures, medications and observations. For diagnoses and procedures, we used the SNOMED clinical vocabulary codes (Donnelly, 2006). The majority of previous studies have used International Classification of Diseases (ICD) codes (Choi et al., 2016a; Li et al., 2020; Rasmy et al., 2021). The decision to use SNOMED was motivated by several factors related to our PHM use case:

1. **Detail and granularity:** SNOMED CT offers a more detailed, granular system, which allows for greater specificity and detail in medical records. It's particularly useful for recording clinical details about diseases, findings, procedures, etc., that might not be adequately captured by ICD.
2. **Clinical care:** SNOMED CT was specifically designed for use in clinical care, making it more effective for EHR systems, clinical decision support systems, and other healthcare IT applications. It can help enhance the quality and effectiveness of care by enabling more precise communication and decision-making.
3. **Interoperability:** SNOMED CT facilitates interoperability between different health information systems and services, making it easier to share, exchange, and understand clinical information.
4. **Research and data analysis:** The greater detail and precision of SNOMED CT can enhance research and data analysis capabilities, contributing to more meaningful health outcomes research and improving public health surveillance.
5. **Comprehensive coverage:** SNOMED CT is known for its comprehensive coverage of clinical content, from diseases and symptoms to diagnostics, therapeutics, and preventive measures. This coverage spans multiple medical specialties and disciplines.

For medications, we used the National Drug Code (NDC) Directory codes. For observations, we used the Logical Observation Identifiers Names and Codes (LOINC) codes. Where a test was associated with a categorical outcome such as “positive” or “negative”, the code for the test was concatenated with the code for the outcome (e.g., 10331-6_negative). In order to capture the values associated with observations measured on a numerical scale, we normalized the values associated with each observation between 0 and 1 using min-max scaling. We then divided these normalized values into 10 bins at:


≥0.0<0.1,≥0.1<0.2,…,≥0.9≤1

Prior to scaling, we winsorized the values for each observation at the 1st and the 99th percentile to remove extreme outliers caused by data entry error. This methodology was chosen ahead of binning according to quantiles because it better maintains the shape of the population distribution. It could allow, for example, rare but significant observation values to be preserved as separate tokens, with the majority of the population falling into bins representing “normal” values.

The binned values were then concatenated with the LOINC codes to give a token which represented a code-value combination. For example, the LOINC code for systolic blood pressure (8480-6) was represented by 10 tokens (8480-6_0 through 8480-6_9) which represented a blood pressure reading in the corresponding bin within the population distribution. To ensure that values were binned consistently, we saved the min and max values for each observation code from the train set and used these to normalize the corresponding values in the transfer learn and test datasets. We did not winsorize the transfer learn and test datasets, but instead set outliers to the saved min or max values. This ensured that all datapoints remained between 0 and 1. For the purposes of explainability, these thresholds were then de-normalized after SHAP analysis so clinicians could see the range of values represented by each observation token.

We also created a series of age tokens for each patient (age_0, age_1, age_2,… age_n). For each year during the period covered by the dataset, a single age token was added representing the age of the patient during that year. These fulfilled a dual purpose in our data. The first was to allow a GRU capture the age of the patient at each encounter and enable observations to be associated with an age. For example, an observation for high blood pressure at age 55 may be associated with a higher risk of a myocardial infraction than a similar reading at the age of 25. Secondly, these age tokens captured the elapsed time in years between entries in the sequence. For example, if there was a period of time between diagnoses in the record, this time period would be represented by successive age tokens. During pre-training, the Skip-Gram algorithm would associate these diagnoses with ages at or close to onset of the condition. Other methodologies have been shown to be effective for creating time-aware embeddings, for example by concatenating age and other demographic information with code or visit embeddings (Choi et al., 2016a). However, in the interest of explainability, we wanted to ensure that each of our embeddings represented a clearly delineated clinical or demographic concept that would be recognizable to PHM practitioners.

**Figure 1:**
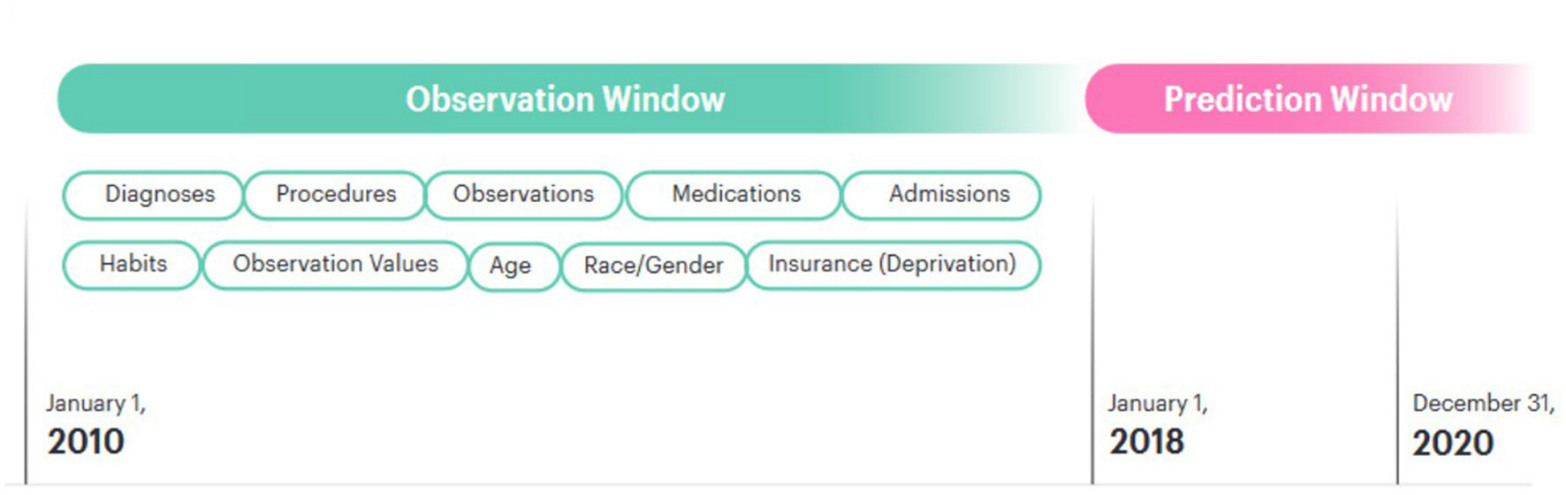
Patient timeline.

For admissions, the type of admission was used described in natural language (e.g., emergency_admission). Our dataset also included information on patient smoking and alcohol consumption taken at intervals throughout their lives. This information was consistently encoded in natural language, for example “smoking_yes”. These tokens were then added to the patient records. In order to add embeddings for gender and race to the model, a token representing each of these concepts was added at the end of the patient record [e.g., (…, race_caucasian, gender_female)]. Our dataset also included information on the type of insurance each patient had. This changed throughout the course of a patient's life, so natural language tokens representing this (e.g., “insurance_private” or “insurance_medicaid”) were added to the patient record with the timestamp when they were updated. In the USA, insurance type is related to a range of socioeconomic, demographic and employment factors which are also determinants of health outcomes (Ross and Mirowsky, 2000; Bittoni et al., 2015; Su et al., 2019; Keisler-Starkey and Bunch, 2020). Insurance information was therefore included with the intention that it should serve as a proxy for these wider determinants. These patient records were ordered sequentially and an array was created for each patient. Figure 1 shows how the patient timelines were constructed.

In EHR data, the sequence of codes within visits is typically not preserved in the data. In line with the recommendations of previous studies, codes were ordered sequentially by date with within-date codes ordered randomly (Choi et al., 2016c). The data was then divided into train, validate and test sets, with 90% of the patients used for training/validation and 5% for testing. The final 5% were held back in order to investigate the value of transfer learning. This ensured no data leakage between the training dataset and the transfer learning data. For training and validation, class 0 patients were down sampled to give a 60:40 ratio of patients who would not develop the disease vs. patients who would. For testing, the model was evaluated on the full test set with the real population distribution.

### 2.4 Feature selection

In order to assess the benefit of the inclusion of novel features and determine the best feature scope for subsequent modeling, a feature selection experiment conducted prior to disease modeling. This study used a combination of three categories of features:

1. Features which have been commonly used in previous studies
  (a) Diagnoses
  (b) Medications
  (c) Procedures
  (d) Observations (history of Laboratory Tests that have been conducted)
2. Features which have been previously used which we included using a different methodology
  (a) Age
  (b) Gender
  (c) Race
  (d) Observation Values (including vital signs and laboratory results)
3. Features we have included for the first time (to our knowledge)
  (a) Admissions
  (b) Insurance type

For details of features included in previous studies, see Table 2. For feature selection, a simple model was created for each disease using a randomly-initialized embedding layer, a global max pooling layer, and a sigmoid output. A baseline model was then trained using a feature set that has been commonly included in previous studies (diagnoses, procedures, medications, and observations). For the baseline model, Observations were represented by LOINC codes without concatenated values. Two experiments were then conducted. The first assessed the performance of each additional feature individually against the baseline model by training a model which included the baseline feature scope plus that feature. The second assessed the cumulative performance by adding the features one by one and retraining each time. The order in which features were added was determined by their novelty, with features for which we have used a different methodology added first, followed by features not previously included. Ten percentage of the training dataset was set aside as a holdout set to evaluate the feature selection experiment. Models were trained using the remainder of the training dataset with a 90:10 train validation split. The results for all four diseases were averaged for each feature set, and the feature set with the highest average performance was chosen for subsequent experiments.

**Table 2:** Summary of features and methodologies used in medical concept representation learning.

**Paper**	**References**	**Clinical**	**Demographic / socioeconomic**	**Methodology notes**
Choi et al. (2016a)	Med2Vec	Diagnoses, procedures, medications, visit embeddings	Age, gender, race	Age, gender, and race represented as demographic vector which was concatenated with visit representation.
Li et al. (2020)	BEHRT	Diagnoses, visit embeddings	Age	Summed concept, age, visit and position embeddings.
Rasmy et al. (2021)	Med-BERT	Diagnoses, visit embeddings	None	Summed concept, visit and position embeddings.
Pang et al. (2021)	CEHR-BERT	Diagnoses, Procedures, and Medications, Visit Embeddings	Age	Created temporal context embeddings by concatenating age and time embeddings with concept embeddings.
Meng et al. (2021)	BRLTM	Diagnoses, procedures, medications, visit embeddings	Age, gender	Summed age and gender embeddings with concept, visit, and position embeddings.
Li et al. (2022)	Hi-BEHRT	Diagnoses, medications, procedures, laboratory tests, drinking status, smoking status, blood pressure, BMI, visit embeddings	Age	Binned BMI and blood pressure according to defined ranges. Summed age embeddings with concept, visit, and position embeddings.
Rupp et al. (2023)	ExBEHRT	Diagnosis, Mmdications, procedures, laboratory tests, drinking status, smoking status, BMI	Age, gender	Stacked all concept and demographic embeddings vertically and summed before passing through the network.
Datta et al. (2022)	Randomly-initialized embeddings with LSTM plus Static Layer	Diagnoses, Medications, Procedures, Vital Signs, Laboratory Results	Age, gender, race	Vital Signs and Lab Results binned according to quantiles (High, Medium High, Medium Low, Low). Age, Gender and Race fed in separately via a static layer.
Grout et al. (2023) (this paper)	Word2Vec (Skip-Gram) embeddings with BiGRU and MaxPooling layer	Diagnoses, medications, procedures, observations (including vital signs and laboratory results), drinking status, smoking status, admissions	Age, gender, race, insurance type	observation values binned according to the described methodology. Age tokens inserted into patient history to represent age at event and time between events. Race and Gender added as tokens at the end of the patient history. Insurance type for each claim inserted into the patient record.

### 2.5 Predictive modeling

We pre-trained a set of embeddings using the full train dataset of 30 m patients. To avoid data leakage, no patient history after the 2018-01-01 cutoff date was included in the pre-training. For the pre-training task, we used the Word2Vec algorithm which was implemented using the Spark MLib library with an embedding dimension of 128 and a window size of 5. In line with the findings of previous research, we used the Skip-Gram implementation (Xiang et al., 2019). Word2Vec was chosen because it has been demonstrated to be effective when pre-training for disease prediction tasks, it is straightforward to implement and was designed with efficiency and scalability in mind (Mikolov et al., 2013). Word2Vec also makes the expansion of data scope easier relative to more sophisticated architectures. For example, BERT can process texts with a maximum length of 512 tokens and scales quadratically with sequence length (Devlin et al., 2018). With all features added added, our patient records had an average length of 511 and we used a max sequence length of 3,000. In real-world contexts, more features relating to wider determinants of health could be available which may further elongate patient records.

**Figure 2:**
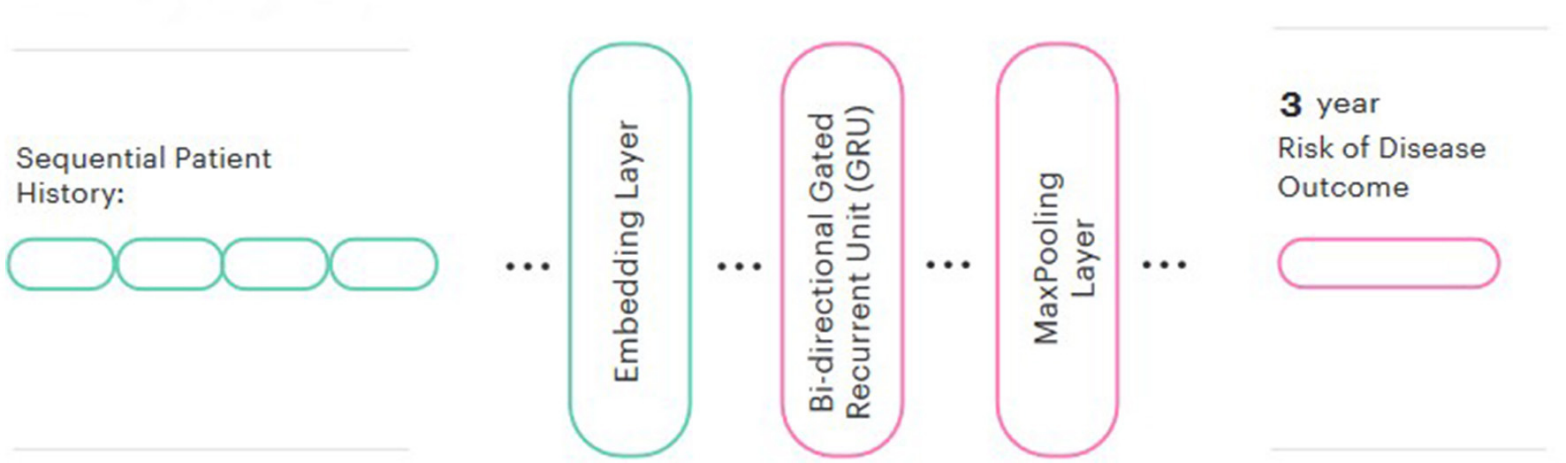
Model architecture.

We then used these pre-trained embeddings to create a predictive model for each disease. In line with the majority of other similar studies (Si et al., 2021), a Bidirectional GRU was chosen for the prediction head. We also added a global max pooling layer and a sigmoid output. We evaluated both frozen and fine-tuned embeddings and compared the performance to a model trained from randomly-initialized embeddings. The Keras library was used for modeling. The final model architecture is represented in Figure 2.

In order to evaluate the effectiveness of transfer learning when limited training samples are available, two experiments were conducted. The first evaluated the within-population benefit of transfer learning using a random sample of 5% of the population set aside for this purpose (the transfer learn dataset). The second experiment evaluated the benefit of transfer learning to a different regional subset of the population. The state of Maryland was selected as the target population for this experiment. Maryland was chosen for several reasons:

1. Maryland contains roughly 5% of the total patients in the dataset (2,358,928).
2. Our dataset has a slight Midwestern bias, and Maryland is geographically separated from the Midwest.
3. Maryland varies demographically from the dataset overall, with a higher African American population and a lower white population.
4. In our dataset, there is less data available per patient in Maryland (average record length 287) than in the rest of the population (average record length 521), making patients more difficult to classify.
5. Disease prevalence is higher in our Maryland sample than in the general population, making proactive intervention desirable.

The differences between the populations are described in Table 3. These figures refer to the patients in our dataset rather than the US population in general. The context (higher disease prevalence but fewer datapoints per patient) was chosen to represent a real-world situation where transfer learning from one population to another could be advantageous.

**Table 3:** Comparison of data dimensions between Maryland (MD) and general population.

**Data dimension**		**Maryland**	**General population (excl. MD)**
Race	White	28.6%	49.2%
	Black or African American	20.4%	8.3%
	Unknown/null	46.2%	36.9%
	Other	4.8%	5.6%
Disease prevalence (onset in prediction window)	Diabetes	0.9%	0.41%
	Hypertension	2.22%	1.54%
	COPD	0.64%	0.32%
	MI	0.32%	0.25%
Medical history	Average patient record length	287	521

For the transfer learning prediction task, diabetes and hypertension were chosen because there were sufficient numbers of disease-positive patients (6000) in the transfer learning samples once cohort selection criteria had been applied. For the first experiment assessing the within-population benefits of transfer learning, the following steps were followed:

1. Random samples were taken from the transfer learn dataset for each disease. These contained between 500 and 6,000 disease-positive patients with a 60:40 ratio between negative and positive patients. The overall training sample sizes therefore ranged from 1,250 to 15,000.
2. Models were trained on each sample using randomly-initialized embeddings and embeddings that had been pre-trained on the train set. An 80:20 train validation split was used.
3. Models were evaluated on the test set which maintained the real-world disease distribution.
4. The process was repeated for a total of three times using different random seeds for sampling. The results were then averaged. This is in line with similar experiments in previous research (Li et al., 2022)

For the second experiment, the following steps were followed:

1. The train and transfer learn datasets were recombined and a new train dataset was created with all patients from the state of Maryland excluded. A new set of embeddings was trained using this dataset.
2. All patients from the state of Maryland were split 70:30 to create new transfer learn and test datasets.
3. The same steps as the first experiment (1–4 above) were repeated using the new embeddings and the Maryland transfer learn and test datasets.

### 2.6 Explainability

To interpret our model, we opted to use SHapley Additive exPlanations (SHAP) (Lundberg and Lee, 2017). SHAP uses a game-theoretic approach to estimate importance values for each model feature. These SHAP values represent the extent to which a particular feature contributes to the model's final prediction. More specifically in our case they correspond to how the prediction would change if we were to remove a particular term from the medical record. This allows us to look at which associated symptoms, procedures, diagnoses and habits were more influential in the patients predicted diagnosis—if a term is particularly important there will be a large change in model prediction when it is removed. Our intention was to develop an approach that would ultimately enable the model's predictions to be interrogated by clinicians to understand the drivers of predictions at an individual or cohort level. SHAP was chosen because it has been the most widely used explainable AI (XAI) technique for similar use cases (Loh et al., 2022).

We implemented the SHAP analysis using the open-source python package according to the documentation (Lundberg and Lee, 2021). For clinical validation, we analyzed 5,000 patients from the validation data for each model. In order to understand which features had the greatest influence on disease prediction at the population level, we summed the SHAP scores and examined the top clinical features (diagnoses, procedures, observations/values, medications). We chose this measure because it accounts for the magnitude of the effect of a feature as well as its prevalence in the population. SHAP values are additive, so this metric represents the overall contribution of a feature to the prediction of disease within the population. We then validated these features with a clinical expert (Lundberg and Lee, 2017). Features for each model were presented to a clinician who provided feedback on the clinical relevance of each feature and suggested changes to the training process where necessary. These changes were then implemented, the model was retrained, and the SHAP analysis was re-run. Once the clinician was satisfied with the feature importances, a final SHAP analysis was run in order to report the results. This used a sample of 5,000 patients for each disease from the test set, with the same ratio of positive and negative patients as the train/validation sets (40:60).

## 3 Results

**Figure 3:**
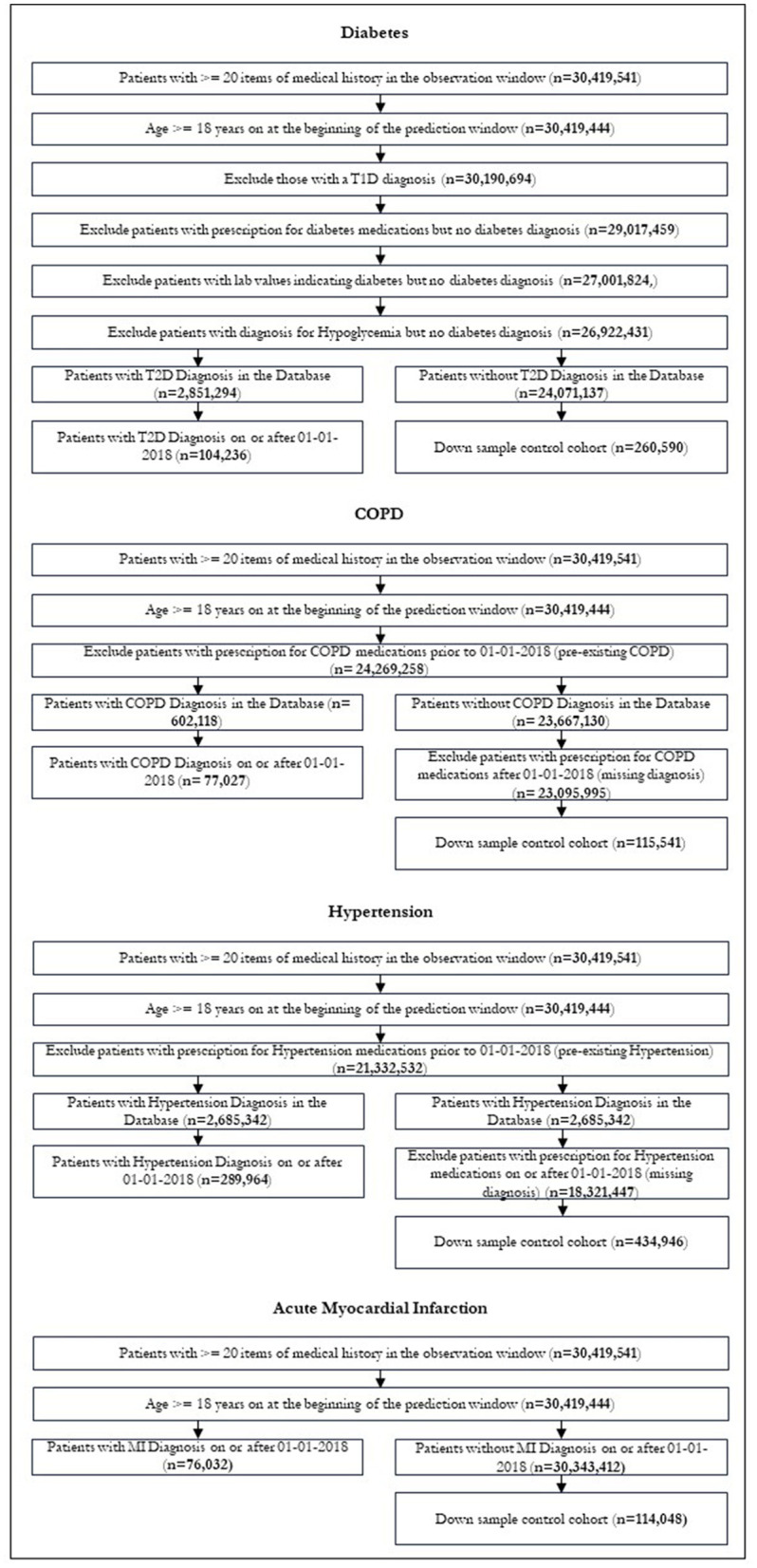
Patient attrition.

After restrictions were applied, we were left with 104,236 patients in our diabetic cohort, 77,027 for COPD, 289,964 for hypertension and 76,032 for MI. Figure 3 shows the patient attrition tables. The flow diagrams in Figure 3 show the patient attrition for each exclusion.

### 3.1 Feature selection

**Figure 4:**
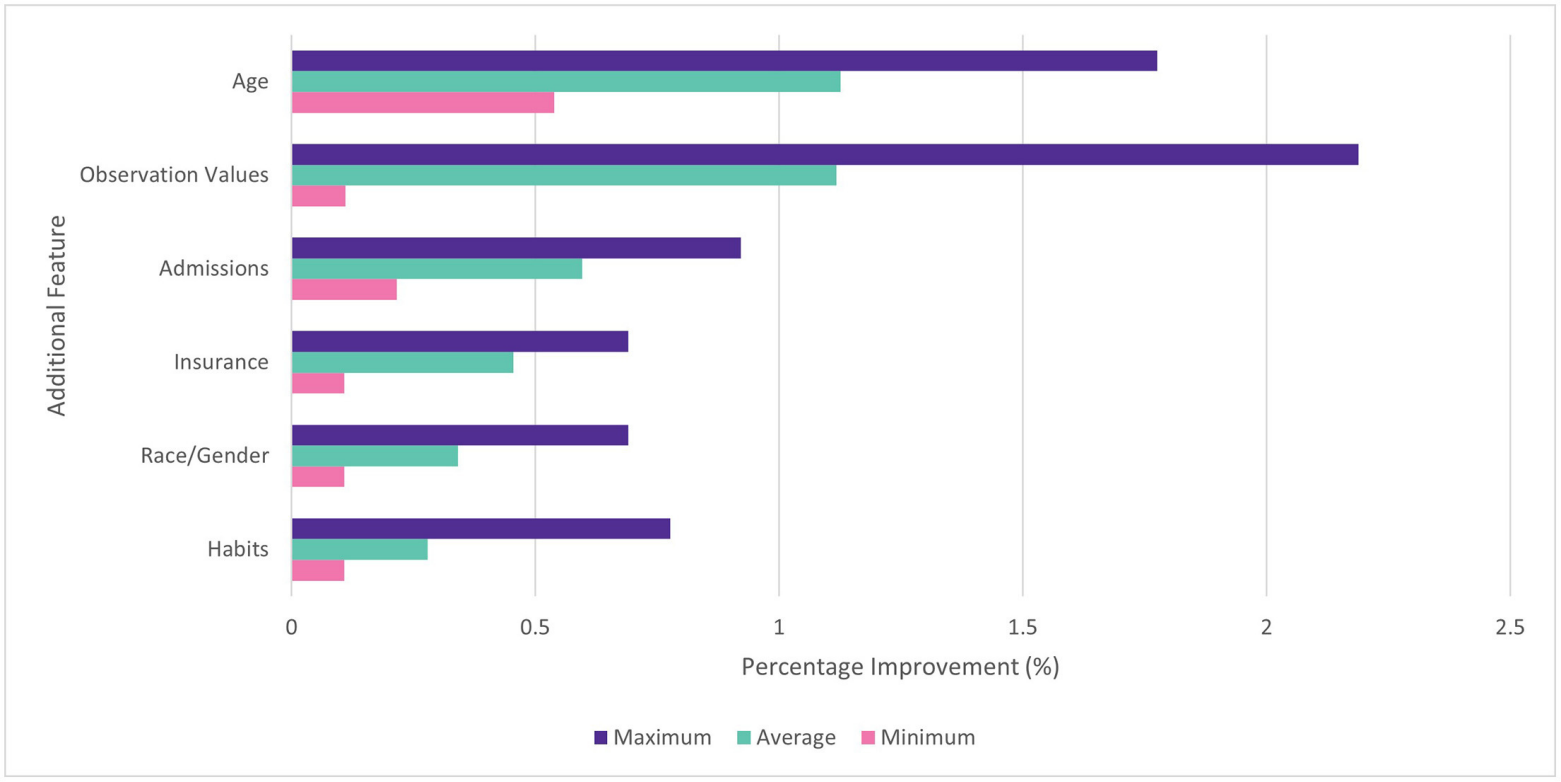
Percentage improvement in ROC AUC score for individual features vs. baseline. Min, max, and average across four diseases.

In the feature selection experiment, it was found that performance increased as more features were added to the dataset. All features were found to improve average performance when added individually and cumulatively. When added individually, addition of age tokens gave the largest average benefit (1.3%) followed by observation values (1.2%), admissions (0.6%), and insurance type (0.5%). The benefit of different features was found to vary by disease. For example, habits (drinking / smoking status) made relatively little impact on the ROC AUC for diabetes (0.1%), but made a larger difference for COPD (0.8%). This is consistent with the connection between COPD and tobacco use. Observation values made the largest impact with the diabetes model (2.2%) and the least with COPD (0.1%). The min, max and average improvements for each feature are shown in Figure 4.

**Figure 5:**
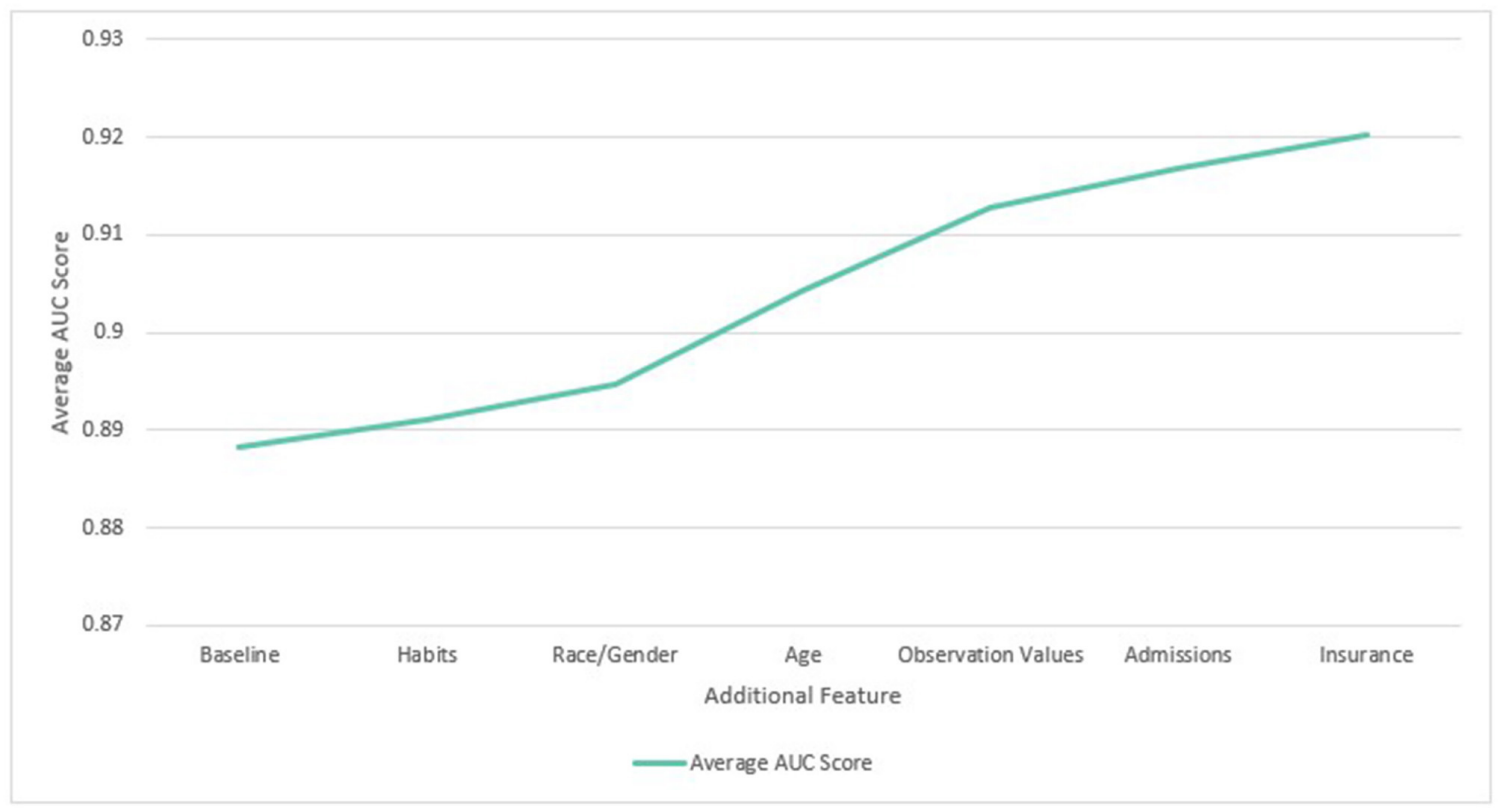
Average ROC AUC score across four diseases with cumulative additional features.

When features were added cumulatively, the average ROC AUC increased from 0.888 to 0.920 (3.6%) relative to the baseline models across diabetes, COPD, hypertension and MI. Age (1.1%) and observation values (0.9%) also gave the largest average benefit. The results are shown in Figure 5. Since all features showed an improvement to performance, they were all included in subsequent experiments.

### 3.2 Pre-training

In line with previous findings, our Word2Vec algorithm was successful at learning the similarity of related medical concepts. Our vocabulary size (157,145) was the largest of any similar study to our knowledge, due to the increased data scope and the separation of observations into multiple binned code-value combinations. We did not conduct a comprehensive analysis of clinical code groupings learned via Word2Vec as this has been conducted in other studies (Choi et al., 2016c). However, we did conduct spot checks on key diseases relevant to our use case. Table 4 shows the most closely related concepts to the diseases we predicted by cosine similarity.

**Table 4:** Predicted diseases: related concepts by cosine similarity.

**Disease**	**Rank**	**Similarity**	**Description**
Diabetes	1	0.91	Type II diabetes mellitus uncontrolled
	2	0.82	Hyperlipidemia
	3	0.82	Polyneuropathy due to diabetes mellitus
	4	0.80	Chronic kidney disease stage 2
	5	0.80	Type 1 diabetes mellitus
	6	0.80	Disorder of nervous system due to diabetes mellitus
	7	0.79	Disorder of nervous system due to type 2 diabetes mellitus
	8	0.78	Disorder due to type 2 diabetes mellitus
COPD	1	0.88	Emphysematous bronchitis
	2	0.86	H/O: asbestos exposure
	3	0.86	Dependence on enabling machine or device
	4	0.84	H/O: pneumonia
	5	0.84	Bronchiectasis
	6	0.81	Acute exacerbation of chronic obstructive airways disease
	7	0.81	Chronic asthmatic bronchitis
	8	0.79	Extreme obesity with alveolar hypoventilation
Hypertension	1	0.92	Dyslipidemia
	2	0.71	Ezetimibe 10 MG oral tablet [Zetia]
	3	0.70	Valsartan 320 MG oral tablet [Diovan]
	4	0.70	Irregular heart beat
	5	0.68	Restless legs
	6	0.67	Hypertriglyceridemia
	7	0.67	Benicar
	8	0.66	Patient counseling education
Acute myocardial infarction	1	0.93	Acute myocardial infarction of anterior wall
	2	0.86	Acute myocardial infarction of lateral wall
	3	0.86	Acute myocardial infarction of anterolateral wall
	4	0.85	Acute myocardial infarction of inferolateral wall
	5	0.85	Coronary arteriosclerosis
	6	0.84	Acute myocardial infarction of inferior wall
	7	0.84	Preinfarction syndrome
	8	0.84	Acute subendocardial infarction

The Word2Vec algorithm was able to categorize the demographic and socioeconomic features successfully as related concepts and map the increasing distance between ages. Race tokens were effectively classified, with the most similar tokens for “Race Caucasian” comprising the other racial groups in our dataset. Gender male and female tokens were also most closely related, with a similarity of 0.96. Interestingly, the tokens for insurance type and race were classified as closely related, which may reflect the real-life correlation between these features (Keisler-Starkey and Bunch, 2020). These are shown in Table 5.

**Table 5:** Wider determinants: related concepts by cosine similarity.

**Concept**	**Rank**	**Similarity**	**Description**
Age—45	1	0.90	Age 44
	2	0.89	Age 46
	3	0.86	Age 47
	4	0.86	Age 43
	5	0.81	Age 42
	6	0.80	Age 48
	7	0.73	Age 41
	8	0.72	Age 49
	9	0.67	Age 50
	10	0.65	Age 40
Race—Caucasian	1	0.98	Asian
	2	0.98	Other
	3	0.98	African American
	4	0.98	Unknown
	5	0.97	Hispanic/Latino
Insurance type—Medicaid	1	0.86	Other public insurance
	2	0.81	Private insurance
	3	0.80	Selfpay
	4	0.77	Race: African American
	5	0.76	Race: Hispanic/Latino
	6	0.76	Race: other
	7	0.74	Race: null
	8	0.74	Race: unknown

Similar laboratory values were also grouped together. For example, the most similar codes to “8480-6_3”, which represented systolic blood pressure between 178.3 and 189, contained other codes representing systolic and diastolic blood pressure observations. Tokens representing different BMI values were also related, as shown in Table 6.

**Table 6:** Observation values: related concepts by cosine similarity.

**Observation/ Value**	**Rank**	**Similarity**	**Description**
Systolic blood pressure, 178.30–189	1	0.94	Systolic blood pressure, 167.60–178.30
	2	0.89	Systolic blood pressure, 156.90–167.60
	3	0.86	Systolic blood pressure, 146.20–156.90
	4	0.78	Diastolic blood pressure, 101.20–108.00
	5	0.73	Systolic blood pressure, 135.50–146.20
	6	0.70	Diastolic blood pressure, 94.40–101.20
	7	0.61	Diastolic blood pressure, 87.60–94.40
	8	0.59	Systolic blood pressure, 124.80–135.50
Body mass index BMI (Ratio), 27.26–30.98	1	0.69	Body mass index BMI (Ratio), 30.98–34.70
	2	0.66	Body mass index BMI (Ratio), 23.54–27.26
	3	0.62	Body mass index BMI (Ratio), 34.70–38.41
	4	0.58	Body weight, 87.82–98.89
	5	0.58	Body mass index BMI (Ratio), 38.41–42.13
	6	0.56	Body weight, 76.75–87.82
	7	0.54	Body mass index BMI (Ratio), 19.82–23.54
	8	0.54	Body weight, 65.68–76.75

Where we have innovated in creating tokens, therefore, it seems that Word2Vec pre-training has succeeded in representing their meaning in relation to other concepts, although further investigation would be needed to fully establish this.

### 3.3 Predictive modeling

Embeddings pre-trained using Word2Vec were found to outperform models training from scratch for all diseases when the embedding layer was fine-tuned. When the embedding layer was frozen, training from scratch from randomly-initialized embeddings was more successful for 3 out of 4 diseases, with COPD being the exception. The results are shown in Table 7:

**Table 7:** Model performance metrics (ROC AUC score).

**Disease**	**Embedding method**	**Randomized embeddings**	**Word2Vec embeddings**
Diabetes	Frozen	N/A	0.9084
COPD	Frozen	N/A	0.9323
Hypertension	Frozen	N/A	0.9122
MI	Frozen	N/A	0.9411

Diabetes	Fine-Tuned	0.9137	0.9166
COPD	Fine-Tuned	0.9314	0.9359
Hypertension	Fine-Tuned	0.9173	0.9203
MI	Fine-Tuned	0.9415	0.9437

Table 8 shows the recall and precision for the top 1, 5, and 10% highest risk cohorts identified by each model.

**Table 8:** Summary of precision and recall by risk population.

**Disease**	**Incidence rate**	**Risk population**	**Precision (%)**	**Recall (%)**
Diabetes	0.43%	1%	9.9%	22.9%
		5%	4.7%	54.6%
		10%	3.0%	70.7%
COPD	0.33%	1%	8.7%	26.5%
		5%	4.2%	63.5%
		10%	2.7%	80.5%
Hypertension	1.56%	1%	32.5%	20.8%
		5%	16.5%	52.9%
		10%	11.1%	71.2%
MI	0.25%	1%	9.4%	38.2%
		5%	3.3%	67.9%
		10%	2.0%	81.5%

**Figure 6:**
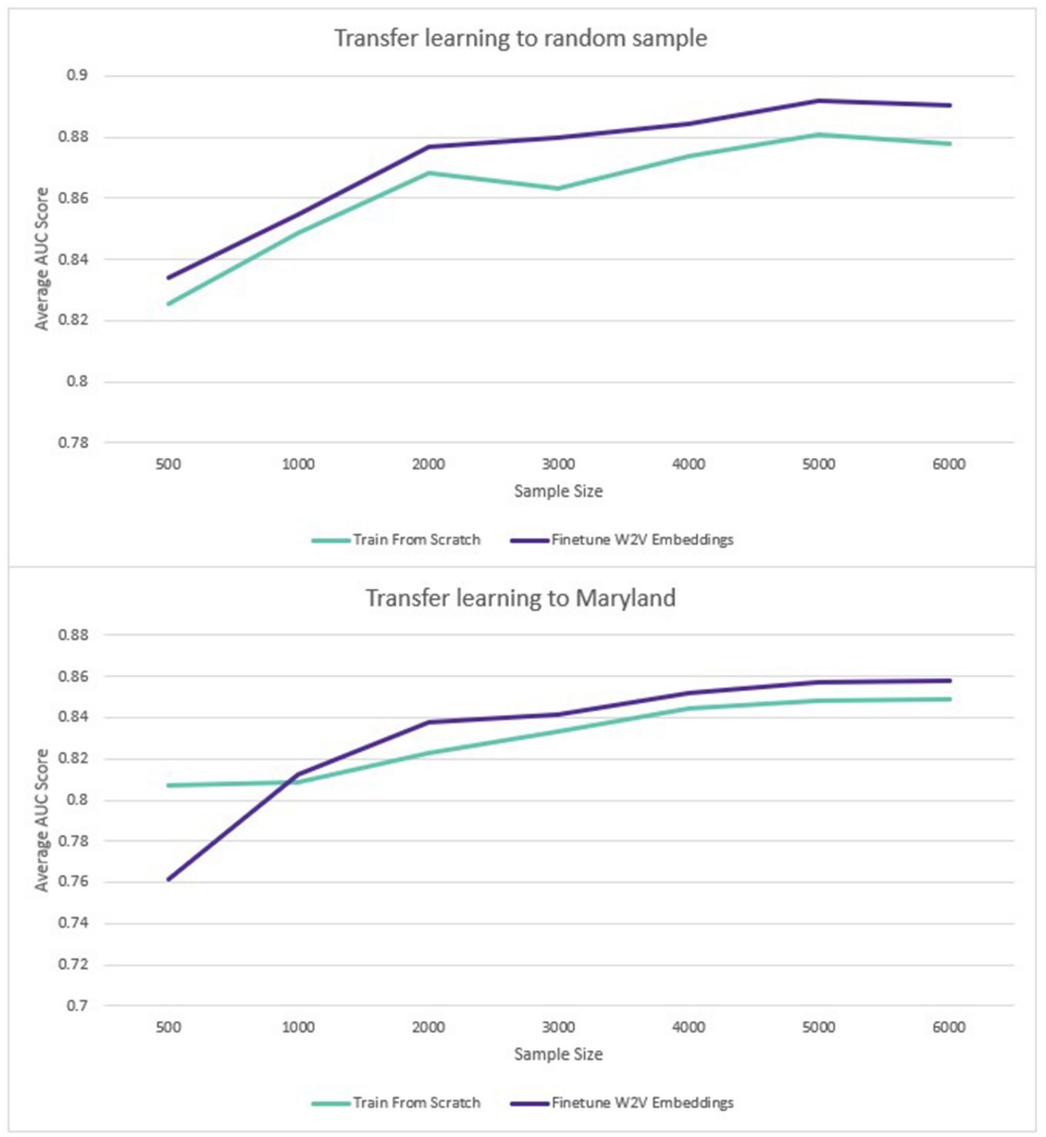
Transfer learning: average ROC AUC score for different sample sizes (diabetes and hypertension).

For transfer learning to sampled datasets containing less labeled data, the Word2Vec embeddings also performed better. The benefit of transfer learning was larger where the pre-training and transfer learning datasets came from the same population (1.2%). Where embeddings trained on data from the rest of the United States were used to predict disease in Maryland, there was also an average benefit to using the pre-trained embeddings but the average benefit across all sample sizes was smaller (0.1%). The results are displayed in Figure 6:

### 3.4 Explainability

**Figure 7:**
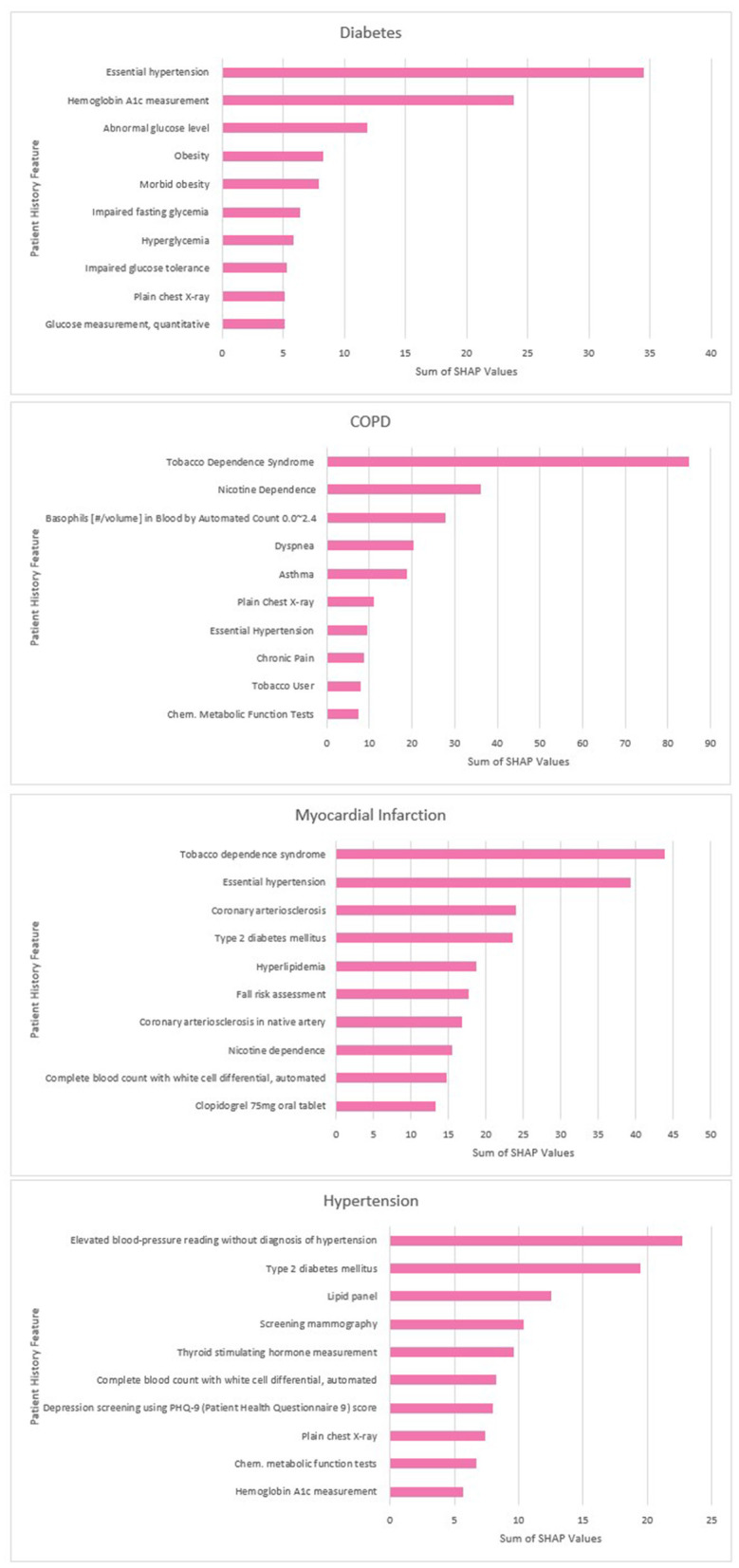
Disease top contributors by summed SHAP values.

The top 10 predictors for each disease according to the sum of the SHAP values are displayed below in Figure 7.

## 4 Discussion

### 4.1 Feature selection

Our study showed that increasing the scope of data included in patient histories to include both lab values and information on wider determinants of health has the potential to improve the performance of disease prediction models. The methodology of binning the values of clinical observations was found to be an effective method of capturing these values for predictive modeling. Across all conditions tested, the addition of these binned values was found to increase the performance of the model. In this study, 10 bins were chosen because they offered superior performance over five bins when tested. The approach we used has the advantage of being straightforward to implement across a large feature set without expert clinical knowledge. If a similar process was put into real-world use, there would be the option to allow clinicians to set the thresholds for binning rather than relying on the population minimum and maximum. This could potentially help reduce the number of tokens for embeddings training if, for example, if it were determined on clinical advice that any values within a given range could simply be grouped into an “expected” or “normal” category. A similar approach of including LOINC codes concatenated with indicators of normality or abnormality has been tested previously and shown to be effective (Rossi et al., 2019). It would be an interesting avenue for future research to assess whether utilizing clinical thresholds of normality vs. abnormality would outperform binning according to the population distribution. It also remains to be tested how well this approach would work when fine-tuning across national populations; for example, whether embeddings trained on a US population would be effective when transfer learning to a European population.

The use of tokens created to represent age at different stages of the patient record was found to improve performance. Similarly, the addition of race and gender tokens at the end of the patient record were a simple but effective way of adding demographic information. A Skip-Gram pre-training task was able to successfully map these related concepts as similar vectors. Other methodologies for adding this information have been proposed. For example, Choi et al. (2016a) concatenated a vector containing demographic information with each visit embedding in their Med2Vec model. Our methodology has two potential advantages. Firstly, it is very straightforward to implement whilst improving the predictive performance of models. It does not require modification of the Spark MLib Word2Vec class, which would simplify the productionization and maintenance of solutions for healthcare providers. Secondly, it maintains the delineation of medical and demographic concepts which may have benefits for the explainability of the algorithm. One single embedding vector represents a single diagnosis, procedure, lab value, age, gender etc. which means, for example, that a SHAP force plot of a patient record could be more straightforward for clinical experts to understand and evaluate (Lundberg and Lee, 2023).

Out of the demographic factors included, age was the most beneficial to model performance. This may be because static demographic features may be inferred from the medical history of the patient. For example, using our methodology we found it possible to “predict” the gender from their constructed history with an accuracy of 98% and a ROC AUC of 0.997 due to the prevalence of sex-specific information in medical records. Race was found to be less predictable although still better than chance. The Mean Absolute Error for “predicting” the current age of a patient given their medical history was 4 years, so it is possible that the performance benefit derived from associating ages with events throughout the medical history. The inclusion of insurance type as a feature was motivated by the hope that this would act as a proxy for social-economic determinants of health. Whilst this is specific to a US context, in other geographies this information could be substituted for other sources of information. For example, indices of deprivation, education, employment status, and housing status may be included.

The predictive influence of the additional feature set was found to vary across diseases in line with medical expectations. For example, the binned observation values were found to provide a greater benefit to diabetes models than age or habits, where measurements such as blood glucose and BMI are key predictors. Conversely for COPD where smoking status over prolonged timescales is a key predictor, habits and age were found to have a larger impact on performance and the addition of laboratory values was less beneficial. We assessed whether the results of this experiment aligned with the findings of the SHAP analysis, and found more observation tokens amongst the top predictors for diabetes, and a greater influence of age and smoking-related features on the model predictions for COPD.

The results of the initial feature selection experiment suggest that the expansion of data scope for the purposes of Population Health Management to include wider determinants of health has the potential to be beneficial. This paper has shown that it is possible to customize and extend clinical vocabularies to encode more predictive information and encompass wider determinants of health outcomes. Our ontology included clinical concepts such as diagnoses and procedures represented by the SNOMED vocabulary but also custom concepts such as age, gender and insurance type which was included for its association with wider socio-economic determinants of health. To our knowledge, this is the first study to combine clinical codes with customized tokens in this way to create embedding representations. In this study, the number of factors we were able to include was limited by the scope of our dataset. However, in real-world implementation more information could be added where this is available to health systems. In several geographies, efforts are underway to create linked datasets which connect medical information to, for example, data from mental health, social and community care, local government and third sector organizations (Tang et al., 2023). The ability to construct more complete histories of the person in context is intended to provide a richer dataset from which to infer the likelihood of health outcomes. The approach outlined in this paper has the potential to provide a flexible, scalable and adaptable methodology for modeling these patient histories in the future.

### 4.2 Predictive modeling

The overall performance of predictive models trained using our methodology was good and comparable to the findings of similar studies. For example, our ROC AUC for predicting the 3-year risk of diabetes (0.92) was in-between that achieved by BEHRT (0.88) and Hi-BEHRT (0.93) in Li et al., although the predictive task was not exactly the same. The Hi-BEHRT authors included type 1 diabetes in their cohort and used a 5 year prediction window (Li et al., 2022). The ROC AUC for predicting the 3-year risk of hypertension (0.92) is comparable to that achieved by Datta et al. (2022) predicting 2-year risk of hypertension with a similar model architecture (0.90). Further research would be required to systematically evaluate the relative performance of our approach to others. It is worth noting that the ideal model for a PHM use case would likely over-predict the number of disease cases. We would hope, for example, that a person who would develop diabetes in 4 or 5 years would be among the at-risk patients even though they would be assigned a 0 label in the training/evaluation data. Likewise, we would want a high risk to be given to a person who would in fact develop diabetes in the next 3 years but whose diabetes would go undiagnosed. It is currently estimated that around 10% of diabetes cases in the USA are undiagnosed (Fang et al., 2022).

In line with previous research, the use of the Skip-Gram implementation of Word2Vec has been found to be an effective pre-training task for learning vector representations of medical concepts (Choi et al., 2016c). When a large training dataset was used, this produced a small average performance increase when compared to training a model from scratch (0.34%). This is in line with the findings of similar research on similarly-sized datasets. For example, Xiang et al. (2019) found embeddings trained via an unmodified Skip-Gram implementation to give a performance increase of 0.49% to the ROC AUC on a predictive task relative to randomly-initialized embeddings when the Skip-Gram embeddings were fine-tuned. We also replicated their finding that Skip-Gram embeddings performed worse than randomly-initialized embeddings without fine-tuning.

When only a limited number of training datapoints were used, the benefit of pre-training was larger, with an average 1.2% increase across all diseases and sample sizes tested. Where the transfer learning sample came from a different sub-population, the benefit was only 0.1%. However, this was affected by the fact that performance on the smallest sample size (500 disease positive patients) had a large negative impact on performance (−6%). For the rest of the sample sizes (1,000–6,000 disease positive patients), the average improvement from pre-training was 1% for the Maryland population, compared to 1.3% for the general population. Although part of the same national population, Maryland was sufficiently distinct demographically to provide a meaningful deviation in model performance. As an additional test to verify this, a model was trained to predict diabetes on the training data from the rest of the US (excluding Maryland). This achieved a ROC AUC of 0.92. When the same model was used to predict diabetes in the unseen Maryland population, the ROC AUC was only 0.83. The potential benefits of transfer learning across populations via pre-trained embeddings are therefore encouraging, especially given the limited research availability of large-scale EHR datasets globally.

Wornow et al. (2023) have suggested that clinical EMR models should be evaluated on tasks which provide more meaningful insight into their utility to health systems. In line with this recommendation, we evaluated the ability of our model to correctly identify at-risk individuals within a large population who could be targeted for preventative interventions. One study with a similar objective used a large dataset (2 m patients) of Administrative Health Data (AHR) in Canada to predict 3-year risk of type 2 diabetes (Ravaut et al., 2021). AHR include some of the same datapoints as EHR (diagnosis history along with some medication and observation history) but are less rich than EHR. The Canadian study chose the recall curve as a key metric for population surveillance—they found that over 40% of the actual diabetes cases were captured within the top 10% of at-risk patients identified by the model, with an overall incidence rate of 0.2%. Our diabetes model offered a substantial improvement on this. Our model was able to capture 55% of actual diabetes cases in the top 5% of at-risk patients and 71% of cases in the top 10% of at-risk patients. The 3-year incidence rate for diabetes in our dataset was 0.4%. Our best-performing model (MI) captured 38% of the actual cases in the top 1% of highest-risk patients identified by the model. This improvement can be explained by the difference in data type and data scope (EHR vs. AHR) as well as modeling approach (Deep Learning vs. XGBoost). It is likely that clinical EMR models trained using similar methodologies to ours would also achieve comparable results. The ability to target healthcare funding efficiently to avert adverse health outcomes is key to PHM. Our models have the potential to support this process by identifying cohorts of high-risk patients who could be supported with proactive care.

### 4.3 Explainability

Our study confirmed that SHAP analysis has the potential to improve the explainability of disease prediction models trained using embedded EHR data. In line with similar research (Datta et al., 2022), our analysis found a combination of established disease predictors and novel features identified by the model. For example, our COPD predictors included tobacco dependence and expected comorbidities (e.g., hypertension) but also features such as chest x-rays which may correlate with likelihood of a COPD diagnosis. The top MI predictors included a fall risk assessment procedure which it is possible was used as a proxy for old age and frailty. This highlights an important limitation of SHAP values; they cannot be used to infer the causes of the outcome itself, only the contribution of the features toward the prediction (Lundberg and Lee, 2017).

Our initial SHAP results for hypertension included some blood pressure medications with high SHAP values that may indicate patients missing a diagnosis code in our dataset. These should have been filtered out by the cohort selection process but had been missed due to data quality issues (e.g., alternative spellings, coding errors, or missing links to medication categories). After consultation with clinicians, these patients were removed from the dataset and the model was retrained. This reduced the validation performance of the model slightly (from 0.924 to 0.922 ROC AUC) as these patients had evidently been easier to classify. Explainability analysis conducted during model development may therefore prevent data quality issues in EHR data from leading to overly optimistic estimates of model performance. Shah et al. (2023) argue that clinical LLMs (including those trained on structured EHR codes) need to be tested in real world deployment and shaped and evaluated by clinicians. This study shows the potential of SHAP analysis to support to this process by enabling clinicians to interrogate the features contributing to model predictions. If this were put into real-world deployment, it is possible SHAP could also be used to allow clinicians to understand the drives of predictions for individual patients and enable clinically-informed judgements about whether a patient predicted as high-risk of a negative outcome would benefit from a preventative intervention.

### 4.4 Limitations

The results of our study highlighted the promise of DL solutions for identifying patients at future risk of disease and providing clinicians with the means to understand and evaluate the drivers of those predictions. However, our study has certain limitations. Firstly, individuals with infrequent interactions with healthcare systems or whose medical histories were unavailable due to attrition would be excluded. Our models were also trained to predict first diagnosis rather than disease onset, which requires patients to come forward for medical assessment with the appearance of symptoms. This may have the combined effect of biasing the performance our models in favor of those who have greater access to medical care. Further work would be required to assess and ensure the fairness of models developed using this methodology across all demographic groups. Secondly, our dataset included only one country, and the generalizability of these results to other geographies was not assessed. It would be valuable to understand whether clinical embeddings pre-trained in one geography could be used to train models in another, and follow-up research is planned to investigate this. Thirdly, our SHAP analysis was run on samples of data with balanced classes to replicate our training methodology. This enables clinical validation of model features, but the distribution of disease onset in real-world populations is highly imbalanced. Recent research found that extreme class imbalance had an adverse effect on the interpretative performance of SHAP when predicting credit defaults using an XGBoost model (Chen et al., 2023). Further research is needed to assess the stability of EHR model explanations on imbalanced datasets before this method could be deployed to enable clinicians on the ground to understand the risk scores for individual patients. Finally, in real-world deployment the prediction window used for training would necessarily be from a period prior to the prediction window for implementation, and there would be a time gap to the training observation window. Multiple factors may have changed including aspects of clinical practice. To assess the real-world performance of these models, a longer-term study would need to be conducted to verify predictions over a future 3-year period.

### 4.5 Conclusions

The DL approach outlined in this study can identify clinically-relevant features from large-scale EHR data and use these to predict future disease outcomes. Expanding the data scope to include binned laboratory values, demographic and lifestyle features was shown to improve performance. Once patient histories and embeddings have been created, it is possible to create multiple disease models quickly using the same methodology, reducing the burden on clinician time required for development. Transfer learning from pre-trained embeddings can also improve performance, especially where limited data is available. The calculation of SHAP values can provide clinicians with a means of evaluating the features that are contributing to the models' predictions. This approach was developed using common python packages and cloud services—technologies which are within the reach of healthcare systems today. The approach presented here is a promising methodology for increasing the precision with which PHM practitioners can identify at-risk patients and take preventative steps to improve their health outcomes.

## Data availability statement

The original contributions presented in the study are included in the article/supplementary material, further inquiries can be directed to the corresponding author.

## Ethics statement

Ethical approval was not required for the study involving humans in accordance with the local legislation and institutional requirements. Written informed consent to participate in this study was not required from the participants or the participants' legal guardians/next of kin in accordance with the national legislation and the institutional requirements.

## Author contributions

RGr: Conceptualization, Investigation, Methodology, Writing—original draft, Writing—review & editing. RGu: Conceptualization, Supervision, Validation, Writing—review & editing. RB: Conceptualization, Project administration, Supervision, Writing—review & editing. ME: Formal Analysis, Investigation, Writing—review & editing. YL: Formal analysis, Investigation, Writing—review & editing. KI: Formal analysis, Investigation, Writing—review & editing. RP: Conceptualization, Methodology, Writing—review & editing. JF: Conceptualization, Methodology, Writing—review & editing. CI: Conceptualization, Funding acquisition, Supervision, Writing—review & editing.
